# Disrupted Practice Effects and Altered Prefrontal Activation in Mild Cognitive Impairment: An fNIRS Study Using the Stroop Task

**DOI:** 10.1002/brb3.70942

**Published:** 2025-11-21

**Authors:** Jae‐Hoon Lee, Kyeongho Byun, Minchul Lee, Min‐Seong Ha

**Affiliations:** ^1^ Department of Sport Science, College of Arts and Sports University of Seoul Seoul Republic of Korea; ^2^ Division of Sport Science; Sport Science Institute & Health Promotion Center, College of Arts & Physical Education Incheon National University Incheon Republic of Korea; ^3^ Department of Sports Medicine, College of Health Science CHA University Pocheon‐si Gyeonggi‐do Republic of Korea; ^4^ Laboratory of Sports Conditioning: Nutrition Biochemistry and Neuroscience, Department of Sport Science, College of Arts and Sports University of Seoul Seoul Republic of Korea

**Keywords:** fNIRS, inhibitory control, mild cognitive impairment, neuroplasticity, prefrontal cortex, Stroop task

## Abstract

**Introduction:**

Executive dysfunction, particularly inhibitory control, is an early core symptom of mild cognitive impairment (MCI) and is often associated with altered prefrontal cortical activity. Repeated engagement in cognitive tasks may offer a means of assessing and potentially enhancing neural adaptability in these regions.

**Objective:**

This study examined how repeated Stroop task performance modulates executive function and prefrontal cortical activation in older adults with MCI, compared to cognitively healthy controls (HC), using functional near‐infrared spectroscopy (fNIRS).

**Methods:**

A total of 37 older adults (17: MCI, 20: HC) completed three consecutive sessions of a computerized color–word Stroop task. Behavioral performance (reaction time) and hemodynamic responses (oxyhemoglobin, HbO) were recorded across predefined prefrontal regions of interest, including the ventrolateral prefrontal cortex (VLPFC), dorsolateral prefrontal cortex, orbitofrontal cortex, and frontopolar cortex.

**Results:**

The results demonstrated a significant improvement in response time and reduced HbO activation in the VLPFC across sessions in the HC group, suggesting enhanced cognitive efficiency and selective inhibition. By contrast, the MCI group demonstrated delayed and limited adaptation, with meaningful changes occurring only in the final session.

**Conclusion:**

Healthy aging is associated with rapid neurofunctional adaptation to cognitive challenges, whereas individuals with MCI exhibit impaired plasticity in executive control circuits. fNIRS sensitively detects early executive deficits, supporting its potential for early diagnosis of cognitive impairment.

## Graphical abstract

In individuals with Mild Cognitive Impairment, repeated cognitive challenges (Stroop Task) fail to induce normal prefrontal cortical adaptation, indicating impaired neuroplasticity in response to cognitive demands.

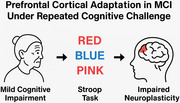

## 1 Introduction

Dementia is a chronic and progressive neurodegenerative disorder that, in its advanced stages, renders individuals incapable of performing basic activities of daily living and imposes substantial physical, psychological, and economic burdens on both patients and caregivers (Gwon 2016). Dementia is often cited as one of the most formidable conditions in older adults. According to data from Statistics Korea (2019), approximately 22.7% of the older population in South Korea is affected by mild cognitive impairment (MCI), a clinical condition widely recognized as a prodromal phase of dementia. This substantial prevalence highlights the urgent need for early diagnostic frameworks and evidence‐based preventive interventions to mitigate dementia progression.

MCI is a transitional cognitive state occurring between normal aging and dementia. Although daily functioning is relatively preserved, individuals with MCI often exhibit objective deficits in specific cognitive domains, such as memory, attention, executive function, and language (Mendez and Cummings 2003). Studies have reported that approximately 12% of individuals with MCI develop Alzheimer's disease annually (Furio et al. 2007), highlighting the urgency of early detection. Beyond hippocampus‐dependent memory decline, MCI is associated with impaired executive function governed by the prefrontal cortex (PFC) (Petersen et al. 1999). These neuropsychological deficits negatively affect self‐esteem, increase depressive symptoms, and lower quality of life (Kim et al. 2012).

While MCI often involves mild cognitive deficits, substantial impairments in executive function, particularly inhibitory control, set shifting, and cognitive flexibility, are frequently observed (Corbo and Casagrande 2022). Among these, selective inhibition can be sensitively assessed using the Stroop task—a well‐established neuropsychological tool that elicits semantic and response conflicts, thereby challenging prefrontal cognitive control systems (Traykov et al. 2007; van Veen and Carter 2005). Neuroimaging studies have demonstrated that Stroop interference engages key areas of the PFC, particularly the ventrolateral prefrontal cortex (VLPFC), which plays a crucial role in suppressing inappropriate responses and maintaining goal‐directed behavior (Aron 2011; Chen et al. 2013).

In healthy older adults, repeated Stroop task performance typically results in faster response times and reduced PFC activation, reflecting improved attentional control and neural efficiency through practice (Davidson et al. 2003; MacLeod 1991).

However, individuals with MCI may exhibit impaired practice effects, operationally defined in this study as the absence of behavioral improvement across repeated blocks of the same cognitive task despite increased task familiarity, accompanied by sustained or even elevated prefrontal activation (Calamia et al. 2012; Duff et al. 2012). In contrast, preserved practice effects are characterized by measurable performance gains across blocks alongside reduced neural activation, indicative of enhanced neural efficiency. The disruption of practice effects in MCI is hypothesized to reflect impaired hippocampal–prefrontal connectivity, which may manifest as persistent or exaggerated prefrontal engagement during task repetition, suggesting inefficiencies in cognitive control mechanisms.

Functional near‐infrared spectroscopy (fNIRS) is a promising neuroimaging tool for older adults owing to its safety, portability, and ability to detect localized prefrontal activation. It is particularly suitable for tracking neuroplastic changes during repeated cognitive tasks (Fan et al. 2025). Nevertheless, most prior studies have focused on healthy populations or employed single‐session Stroop tasks, leaving a gap in the understanding of how repeated task performance affects brain function in MCI.

The present study aims to address a critical gap in the literature by being among the first to systematically examine disrupted practice effects in individuals with mild MCI through the use of a multi‐session Stroop paradigm combined with fNIRS. In contrast to prior studies that predominantly relied on single‐session designs or focused exclusively on behavioral metrics, our methodology integrates longitudinal behavioral assessment with simultaneous hemodynamic monitoring. This design enables a direct investigation of practice‐related neural adaptations and how they diverge between individuals with MCI and cognitively healthy older adults. By concurrently evaluating changes in task performance and associated prefrontal activation across repeated sessions, the study advances current understanding by identifying a potential neurophysiological marker of early executive dysfunction. Furthermore, by explicitly quantifying both intact and disrupted practice effects, the present work introduces a novel conceptual framework for distinguishing between normative and pathological trajectories of cognitive adaptation in aging. Specifically, we hypothesized that healthy individuals would demonstrate reduced activation in the PFC over repeated sessions—indicative of increased neural efficiency in selective inhibition—whereas individuals with MCI would show delayed or attenuated adaptation.

## 2 Methods

### 2.1 Participants

Participants were recruited using purposive sampling targeting older adults aged 65–85 years. Recruitment was conducted through public advertisements, and individuals who voluntarily responded were invited to participate in the study. All participants provided written informed consent after receiving a detailed explanation of the aims and procedures of the study.

MCI was determined based on scores obtained from the Korean version of the Montreal Cognitive Assessment (K‐MoCA). The required sample size was calculated using prior power analysis (G*Power 3.1, Kiel University, Kiel, Germany). Based on a repeated‐measures ANOVA design with a medium effect size (*f* = 0.25), alpha = 0.05, and statistical power (1 − *β*) = 0.90, a minimum of 36 participants was required. To account for potential attrition, 40 participants were initially recruited, with 20 individuals assigned to each group (MCI and healthy controls [HCs]).

Following data collection, three participants were excluded owing to measurement errors and missing values. The final sample included 17 participants with MCI (3 males and 14 females) and 20 cognitively HCs (8 males and 12 females), as presented in Table 1.

**TABLE 1:** Characteristics of participants.

Variables	MCI (*m* = 3, *f* = 14)		HC (*m* = 8, *f* = 12)	
	*M*	SD	*M*	SD
Age (years)	71.17	2.55	71.67	2.74
Height (cm)	153.44	4.87	160.00	9.40
Weight (kg)	58.09	4.79	61.03	10.19
BMI (kg/m^2^)	24.68	1.72	23.83	3.38
Body fat (%)	36.54	4.16	32.23	8.67
MoCA	18.47	2.27	25.15	2.16

*Note*: Values are presented as mean ± standard deviation.

The inclusion criteria required participants to be free from any diagnosed neurological disorders within the previous 6 months and excluded individuals with psychiatric illness or a history of chronic alcohol or substance abuse. Auditory function was assessed using the Weber and Rinne tuning fork test (Kelly et al. 2018), and visual acuity was screened using a Snellen chart. Only the participants with normal hearing and vision were included in the final analysis.

All procedures were approved by the institutional review board of Dongguk University (DUIRB‐202208‐10) and conducted in accordance with the ethical standards outlined in the Declaration of Helsinki.

### 2.2 Study Design

This study adopted a repeated‐measures design to examine changes in prefrontal activation across sessions. The participants were classified into two groups based on their cognitive status: those with MCI and cognitively healthy older adults (HCs).

Prior to the experimental task, all participants underwent a 10‐min resting state period to stabilize the baseline neural activity. They then additionally completed a 3‐min resting‐state measurement for analysis, followed by a computerized color–word Stroop task across three consecutive sessions, with each session consisting of 18 trials including congruent, incongruent, and neutral conditions. During task execution, the brain activity in the PFC was continuously monitored using a portable fNIRS system.

**FIGURE 1:**
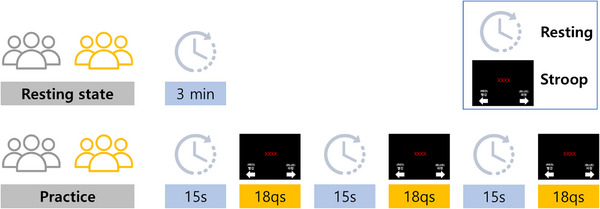
Experimental paradigm of the repeated Stroop task. Participants first completed a 3‐min resting state session to establish baseline neural activity, followed by a brief Stroop task practice. The main task consisted of three repeated Stroop sessions. Each session began with a 15‐s pre‐task rest period (fixation cross), followed by 18 Stroop qs (questions). This structure was repeated across three sessions. Hemodynamic responses in the prefrontal cortex were continuously recorded using a portable functional near‐infrared spectroscopy (fNIRS) system throughout the entire procedure.

The fNIRS device was positioned to capture hemodynamic responses in the PFC regions. Hemodynamic responses were recorded and analyzed to assess task‐related activation and changes in neural efficiency over repeated exposure (Figure 1).

### 2.3 Stroop Task

The color–word matching Stroop task used in this study was originally developed by Stroop (1935) and has since become a widely adopted neuropsychological tool for assessing inhibitory control and attentional regulation. The version employed in the present study consisted of three sessions (blocks), each comprising 18 trials (6 congruent, 6 incongruent, and 6 neutral trials presented in a randomized order), for a total of 54 trials per participant. This experiment was designed using a block design, in which hemodynamic responses were analyzed by comparing the Stroop task blocks to a 3‐min resting baseline period.

**FIGURE 2:**
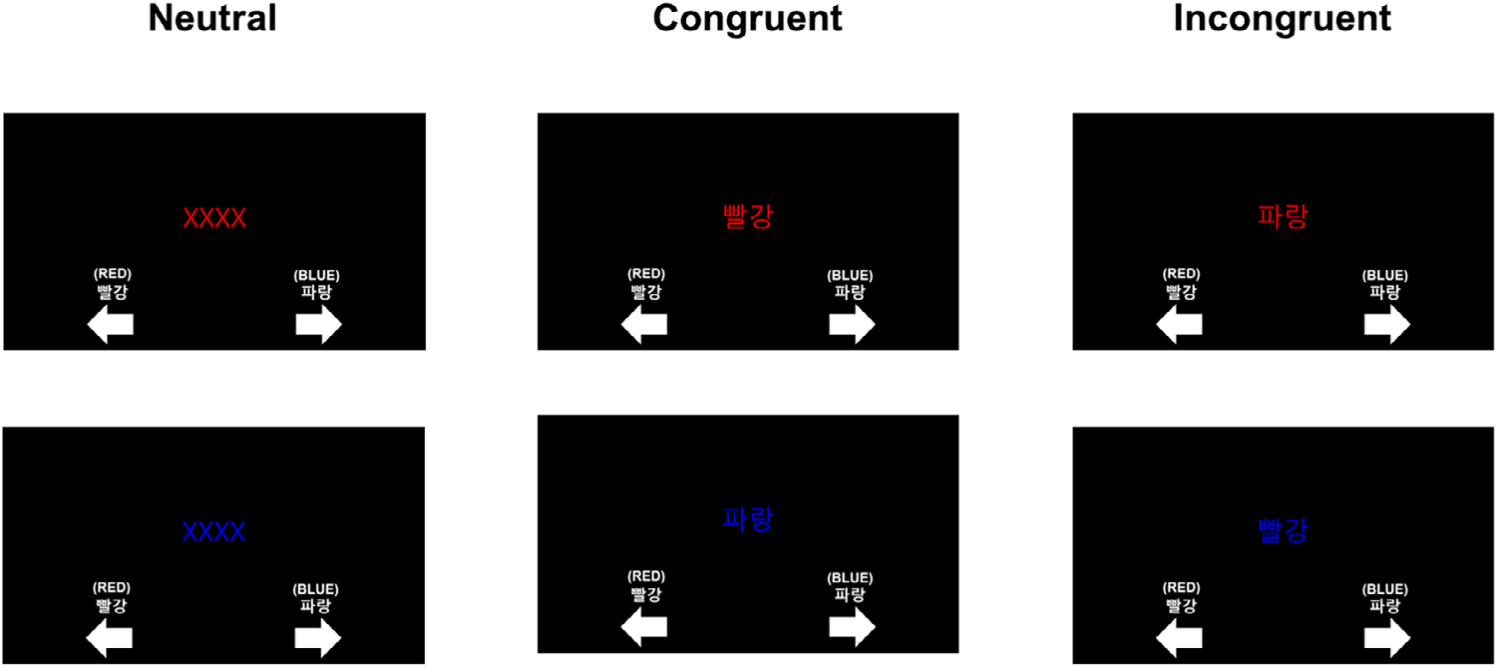
Samples of the three different conditions for the Korean version of the Stroop test: The task consisted of three conditions: neutral (top left), congruent (top center), and incongruent (top right). In the neutral condition, a repeated letter string (“XXXX”) was presented in red or blue, and participants responded based on the font color. In the congruent condition, the color word matched the font color (e.g., “빨강” [red] in red), while in the incongruent condition, the color word and font color did not match (e.g., “파랑” [blue] in red). Below each stimulus, response instructions in both Korean and English were presented, with arrows indicating the appropriate response direction. The task required participants to respond to the font color rather than the word meaning.

In each trial, a single word appeared at the center of a computer screen, and participants were instructed to respond by identifying the color of the text, regardless of the semantic meaning of the word. Responses were made by pressing either the left button (“Red”) or the right button (“Blue”), as appropriate (Figure 2).

The stimuli included the Korean words for “Red” (빨강), “Blue” (파랑), and a neutral letter string (“XXXX”), each displayed in either red or blue font. All the stimuli were presented in Hangul (Korean script). Each stimulus remained on the screen for 3.5 s, and the participants were required to respond within this interval. The task parameters were as follows: nBlock = 3, nTrial = 18, and stimulus duration = 3.5 s.

The task was implemented using a Python‐based script that automatically recorded all behavioral responses, including the reaction time and accuracy for each trial. The fNIRS data were analyzed to assess the differences in prefrontal hemodynamic activity between the resting baseline and the Stroop task blocks, thereby capturing neural correlates of cognitive demand under inhibitory control conditions.

### 2.4 Cognitive Screening: MoCA‐K

The MoCA‐K was used to screen for MCI. The MoCA was originally developed by Nasreddine et al. (2005) and was subsequently adapted to the South Korean population by Lee et al. (2008). The MoCA‐K evaluates multiple cognitive domains, including visuospatial/executive function, language, attention, memory, abstraction, delayed recall, and orientation.

In accordance with the recommendations of Park et al. (2010), a cutoff score of 23 was adopted, which demonstrated a sensitivity of 70% and a specificity of 92% in detecting MCI in older Korean populations. To adjust for the influence of educational attainment, one additional point was added to the total score of participants with ≤ 6 years of education, as per the standard MoCA guidelines.

Based on the adjusted scores, participants scoring ≥ 23 were classified as cognitively normal (HC), whereas those scoring ≤ 22 were identified as having MCI.

### 2.5 fNIRS Data Acquisition and Analysis

To investigate task‐related hemodynamic responses in the PFC during repeated execution of the color–word Stroop task, fNIRS was employed in this study. A portable, wireless, and noninvasive fNIRS device (NIRSIT; OBELAB Inc., Seoul, South Korea) was used to continuously monitor relative changes in oxyhemoglobin (HbO) concentrations, which served as an indirect proxy for underlying neural activity.

**FIGURE 3:**
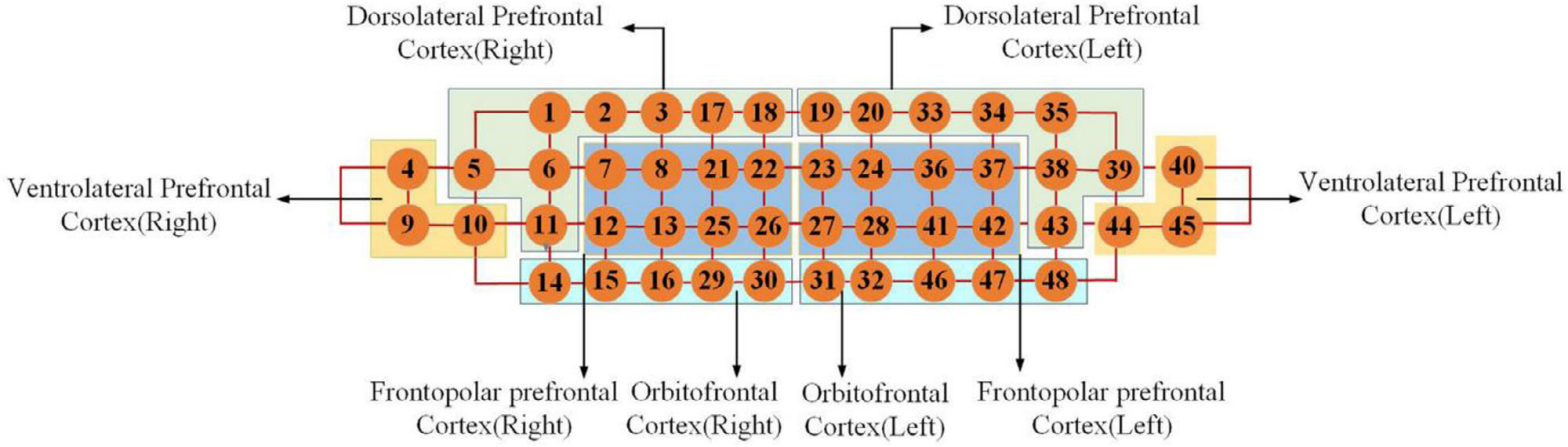
NIRS channel functional area division. From: prefrontal fNIRS‐based clinical data analysis of brain functions in individuals abusing different types of drugs.

The NIRSIT system provides a temporal resolution of 8.138 Hz and utilizes dual‐wavelength near‐infrared light (780 and 850 nm) at power levels below 1 mW to ensure participant safety and maintain signal integrity. The device is equipped with 24 light sources and 32 detectors arranged in a fixed optode geometry, resulting in a total of 48 measurement channels. Each source–detector pair is separated by approximately 3 cm, allowing for effective penetration to a cortical depth of approximately 3 cm. The optode cap was fitted according to the manufacturer's standardized headgear protocol and aligned with the international 10–20 EEG system using four anatomical reference points (Fp1, Fp2, Fpz, and Cz). The bottom edge of the array was positioned just above the eyebrows to ensure consistent coverage of the prefrontal region across participants. The spatial configuration of the channels relative to anatomical landmarks and their corresponding Brodmann areas is illustrated in Figure 3.

Each measurement channel was assigned to one of eight predefined regions of interest (ROIs), comprising the dorsolateral prefrontal cortex (DLPFC), VLPFC, orbitofrontal cortex (OFC), and frontopolar cortex (FPC), bilaterally. This ROI classification was informed by standard cytoarchitectonic definitions (Brodmann 1909) and the manufacturer's brain atlas. While channels were mapped to Brodmann areas for interpretive purposes, it should be noted that fNIRS lacks the spatial resolution to precisely localize cortical structures in the absence of co‐registered MRI data; thus, these anatomical assignments should be interpreted as approximations.

To minimize potential sources of noise, all measurements were conducted under controlled lighting conditions with ambient fluorescent light eliminated. Prior to task onset, a calibration procedure was performed to ensure uniform signal quality across channels.

The raw optical density data were transformed into relative changes in HbO concentrations using the modified Beer–Lambert law (MBLL; Delpy et al. 1988). Preprocessing was carried out using the NIRSIT PC Tool (v2.8) and the NIRSIT Analysis Tool (v3.7.5) in the following sequence: First, a bandpass filter (0.005–0.1 Hz) was applied to attenuate physiological noise arising from cardiac and respiratory cycles. Next, motion artifacts were corrected using the Temporal Derivative Distribution Repair (TDDR) algorithm (Fishburn et al. 2019). Then, all signal traces underwent visual inspection for abrupt spikes, with manual interpolation performed to preserve signal continuity when necessary. Finally, channels were excluded from analysis if they failed to meet one or more quality control criteria: specifically, channels were rejected if their mean signal intensity was below 30 (Yücel et al. 2021), if the coefficient of variation exceeded 15% (Pfeifer et al. 2018), if 5% or more of the recorded values were consecutively identical, or if an implausible inverse correlation (*r* < −0.9) was observed between HbO and deoxy‐hemoglobin signals (Takizawa et al. 2014).

Following preprocessing, the fNIRS time‐series data were segmented into distinct temporal epochs, including pre‐task baseline, task execution periods, and post‐task recovery. Task‐related neural activity was modeled using a generalized linear model (GLM), enabling the estimation of standardized hemodynamic response amplitudes at both individual and group levels. Analyses were performed separately for each channel and ROI to identify region‐specific activation patterns associated with repeated cognitive control demands.

To facilitate interpretation, spatial activation maps were generated to visualize HbO concentration changes across all 48 channels and ROIs, allowing direct comparison between MCI and HC groups. All statistical analyses were conducted using a predefined significance threshold of *p* < 0.05, and where appropriate, adjustments for multiple comparisons and inter‐channel variability were applied to ensure statistical rigor and validity.

### 2.6 Statistical Analysis

All statistical analyses were performed using IBM SPSS Statistics, version 28.0 (IBM Corp., Armonk, NY). Descriptive statistics, including the mean and standard deviation, were calculated for each variable of interest. For reaction time, the normality of the data distribution was assessed using the Shapiro–Wilk test, and all variables met the assumption of normality, justifying the use of parametric statistical methods. For accuracy, the Shapiro–Wilk test indicated that the normality assumption was violated; therefore, a nonparametric Friedman test was employed to examine differences across sessions within each group. When the Friedman test revealed statistical significance, pairwise comparisons were conducted using the Wilcoxon signed‐rank test with Bonferroni correction to control for type I error.

To examine within‐group differences across repeated Stroop task sessions, a one‐way repeated‐measures analysis of variance (RM ANOVA) was conducted separately for each group (MCI and HCs). Additionally, to assess the effects of group (MCI vs. HC), time (Sessions 1–3), and their interaction, a 2 × 3 two‐way repeated measures analysis of variance (ANOVA) was employed. When significant main or interaction effects were observed, post hoc pairwise comparisons were conducted using the Bonferroni correction to control for type I error. All statistical tests were two‐tailed, and the level of significance was set at α = 0.05.

## 3 Results

### 3.1 Stroop Response Time

**FIGURE 4:**
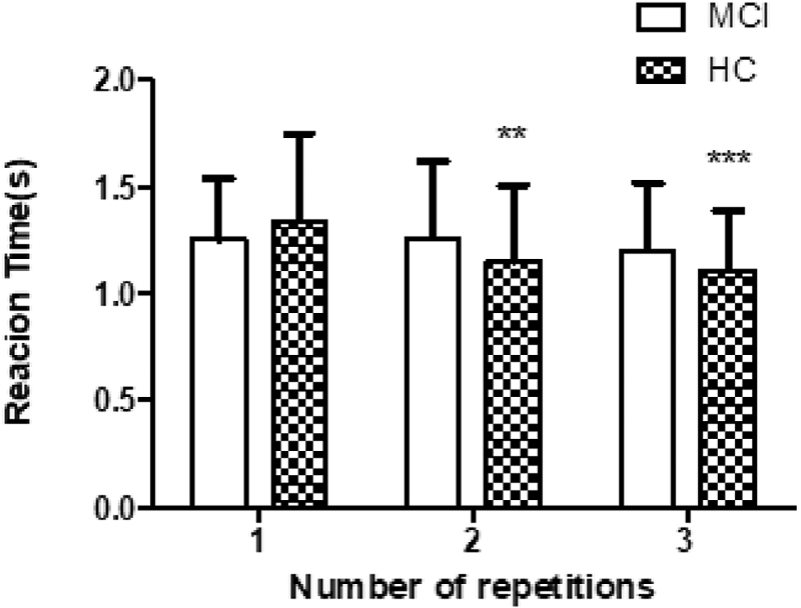
Changes in reaction time over repeated Stroop task sessions between groups: A significant main effect of time (*F* = 7.095, *p* = 0.002), as well as a significant interaction between time and group (*F* = 3.864, *p* = 0.026), was demonstrated, while the main effect of group was not significant. Post hoc analysis revealed that the healthy control (HC) group demonstrated a significant reduction in reaction time in Sessions 2 (*p *< 0.01) and 3 (*p* < 0.001) compared to Session 1, indicating cognitive adaptation or practice effects. In contrast, the mild cognitive impairment (MCI) group did not demonstrate significant changes in reaction time across sessions.

The analysis of response times on the repeated Stroop task across the three sessions for older adults with MCI and cognitively HC is summarized in Figure 4.

A significant main effect of time was observed, indicating that the response times varied across the three task sessions (*F* = 7.095, *p* = 0.002). Furthermore, the interaction between group and time was statistically significant (*F* = 3.864, *p* = 0.026), suggesting that changes in response time over the sessions differed between the two groups. However, the main effect of the group was not significant, indicating no overall difference in the average response times between the MCI and HC groups across all sessions. Post hoc analyses using Bonferroni correction revealed that the HC group demonstrated a significant improvement in response time; specifically, participants demonstrated significantly faster responses in Sessions 2 (*p* < 0.01) and 3 (*p* < 0.001) than in Session 1. In contrast, the MCI group did not exhibit any significant differences in response times across the three sessions. These findings suggest that healthy older adults experienced a measurable practice or cognitive adaptation effect across repeated Stroop task performance, while individuals with MCI failed to demonstrate significant gains, indicating impaired behavioral plasticity in response to repeated cognitive challenges.

### 3.2 Stroop Accuracy Rate

**FIGURE 5:**
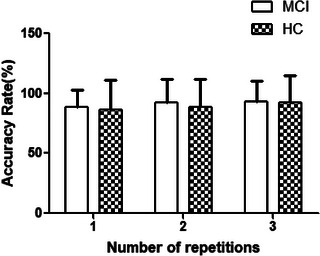
Changes in accuracy rate over repeated Stroop task sessions between groups: A nonparametric Friedman test revealed no significant differences in accuracy across the three Stroop task sessions for the mild cognitive impairment (MCI) group (*χ* ^2^(2) = 4.84, *p* = 0.089). In contrast, the healthy control (HC) group demonstrated a significant main effect of time (*χ* ^2^(2) = 7.75, *p* = 0.021). Post hoc analysis with Bonferroni correction indicated no significant difference between Sessions 1 and 2 (*p* = 0.574), while accuracy in Session 3 showed a trend toward improvement compared with both Sessions 1 (*p* = 0.053) and 2 (*p* = 0.081), although these did not reach the corrected significance threshold. These results suggest that, alongside faster responses, the HC group exhibited a tendency toward higher accuracy with repeated Stroop task performance, indicating potential enhancement of selective attention. Conversely, the MCI group failed to demonstrate notable gains in accuracy, underscoring limitations in executive function adaptability.

The analysis of accuracy rates on the repeated Stroop task across the three sessions for older adults with MCI and cognitively HCs is summarized in Figure 5.

In contrast to the response time analysis, accuracy data did not meet the assumption of normality; therefore, the nonparametric Friedman test was employed. For the MCI group, no significant differences in accuracy were found across the three Stroop task sessions (*χ* ^2^(2) = 4.84, *p* = 0.089). In the HC group, however, accuracy differed significantly between sessions (*χ* ^2^(2) = 7.75, *p* = 0.021). Post hoc analysis with Bonferroni correction revealed no significant difference between Sessions 1 and 2 (*p* = 0.574), but accuracy in Session 3 showed a trend toward improvement compared with Sessions 1 (*p* = 0.053) and 2 (*p* = 0.081), although these did not reach the corrected significance threshold. These findings indicate that, in addition to faster responses, the HC group exhibited a tendency toward higher accuracy with repeated Stroop task performance, suggesting enhanced selective attention. In contrast, the MCI group failed to demonstrate notable gains in accuracy, further highlighting limitations in executive function adaptability.

### 3.3 Cortical Activation

The analysis of task‐related prefrontal activation during repeated Stroop task performance, stratified by Brodmann's area, is presented in Table 2. A series of two‐way repeated‐measures ANOVAs were conducted to examine the effects of group (MCI vs. HC) and time (Sessions 1–3) on HbO concentration across the subregions of the PFC.

**TABLE 2:** Changes in Brodmann areas over time.

Brodmann area	Group	Practice 1	Practice 2	Practice 3	*F*‐value (*p*)		*ηp* ^2^
Left DLPFC	MCI	0.0016 ± 0.0407	−0.0100 ± 0.037	−0.0086 ± 0.0282	time	2.777 (0.070)	0.085
					group	3.639 (0.066)	0.057
	HC	0.0360 ± 0.0480	0.0239 ± 0.0596	0.0126 ± 0.0617	time × group	0.525 (0.594)	0.017
Right DLPFC	MCI	−0.0174 ± 0.0814	−0.0133 ± 0.0577	0.0010 ± 0.0637	time	1.291 (0.282)	0.041
					group	4.761 (0.037)	0.074
	HC	0.0381 ± 0.0442	0.00694 ± 0.0323	0.0249 ± 0.3920	time × group	1.623 (0.206)	0.051
Left FPC	MCI	0.0127 ± 0.0406	0.0051 ± 0.0472	0.0109 ± 0.0275	time	1.004 (0.372)	0.030
					group	3.821 (0.059)	0.056
	HC	0.0409 ± 0.0452	0.0262 ± 0.0498	0.0287 ± 0.0413	time × group	0.222 (0.802)	0.007
Right FPC	MCI	0.0099 ± 0.0608	0.0107 ± 0.0405	0.0075 ± 0.0524	time	0.834 (0.439)	0.025
					group	0.264 (0.611)	0.004
	HC	0.0274 ± 0.0382	0.0098 ± 0.0042	0.0123 ± 0.0441	time × group	0.754 (0.475)	0.023
Left OFC	MCI	0.0459 ± 0.0486	0.0430 ± 0.0486	0.0426 ± 0.0293	time	1.431 (0.247)	0.044
					group	0.152 (0.700)	0.002
	HC	0.0631 ± 0.409	0.0427 ± 0.0618	0.0419 ± 0.0441	time × group	0.778 (0.464)	0.024
Right OFC	MCI	0.0143 ± 0.0806	0.0040 ± 0.0686	0.0268 ± 0.0514	time	1.361 (0.263)	0.037
					group	0.102 (0.751)	0.002
	HC	0.0437 ± 0.0838	0.0168 ± 0.0755	0.00437 ± 0.0806	time × group	2.591 (0.082)	0.069
Left VLPFC	MCI	−0.0247 ± 0.0854	0.00761 ± 0.0733	−0.0207 ± 0.067**	time	0.669 (0.517)	0.025
					group	0.549 (0.465)	0.010
	HC	0.0312 ± 0.0428***	−0.0148 ± 0.0735*	−0.0045 ± 0.0849	time × group	4.067 (0.023)	0.135
Right VLPFC	MCI	0.0026 ± 0.1170	−0.0250 ± 0.1050	−0.0458 ± 0.131	time	5.072 (0.009)	0.150
					group	0.700 (0.410)	0.012
	HC	0.0344 ± 0.0496	−0.0125 ± 0.0721*	−0.0183 ± 0.0789*	time × group	0.190 (0.827)	0.007

*Note*: Values are presented as mean ± standard deviation.

Abbreviations: DLPFC, dorsolateral prefrontal cortex; FPC, frontopolar cortex; HC, healthy cognition; MCI, mild cognitive impairment; OFC, orbitofrontal cortex; VLPFC, ventrolateral prefrontal cortex.

* p < 0.05 vs. Practice 1.

** p < 0.05 vs. Practice 2.

*** p < 0.05 vs. MCI.

**FIGURE 6:**
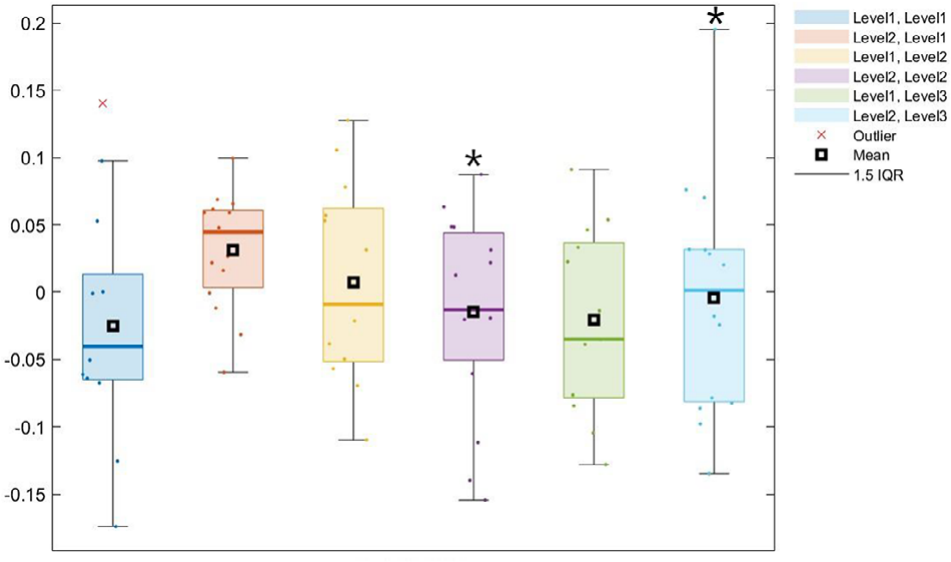
Changes in oxygenated hemoglobin (HbO) levels in the left VLPFC over time between groups: A significant interaction effect was observed (*F* = 4.067, *p* = 0.023). Post hoc analysis revealed that the HC group demonstrated a significant decrease in HbO levels from Sessions 1 to 2 (*p* < 0.05) and significantly higher HbO levels in Session 1 compared to the MCI group (*p* < 0.05). “Level 1 (Group)” refers to the cognitive status, with Level 1 = mild cognitive impairment (MCI) and Level 2 = healthy control (HC). “Level 1 (Session)” refers to the session number.

**FIGURE 7:**
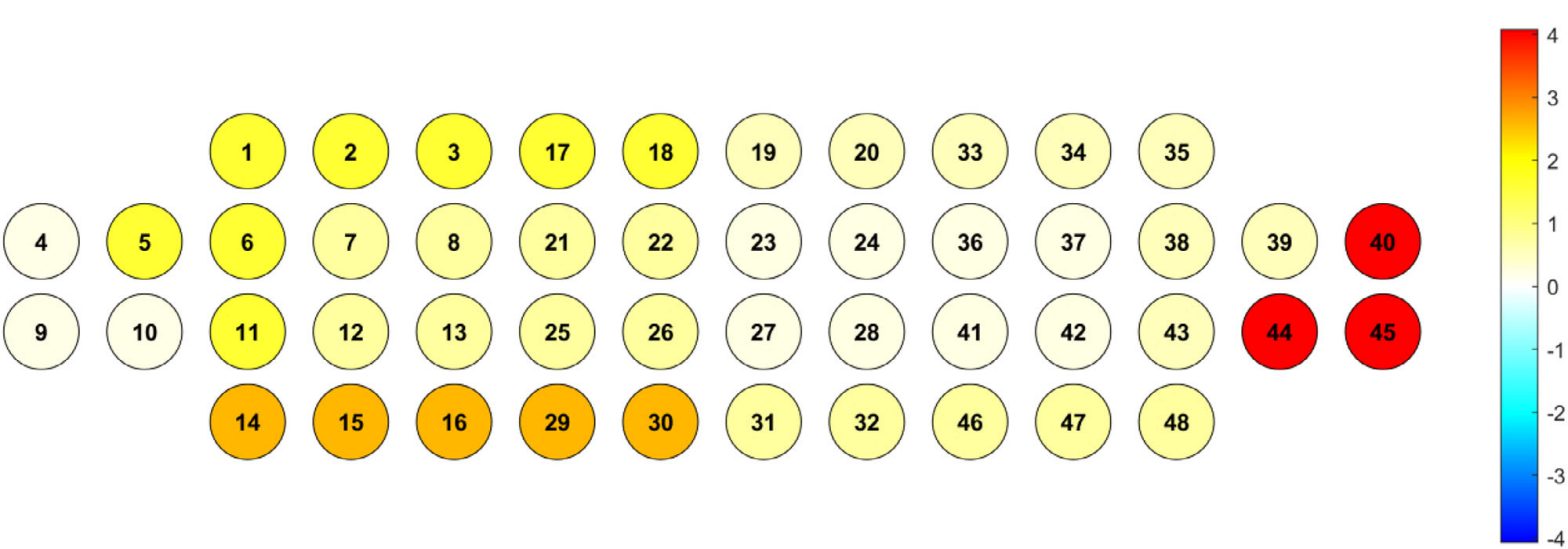
Heat map of group × time interaction effects on oxyhemoglobin (HbO) concentration across prefrontal cortex channels during repeated Stroop task performance: Warmer colors (yellow to red) indicate positive *t* values, reflecting greater HbO changes in the HC group relative to the MCI group over time, whereas cooler colors (light blue to dark blue) indicate negative *t* values, representing greater HbO decreases in the MCI group relative to the HC group. The color scale ranges from −4 (dark blue) to +4 (dark red). Significant interaction effects were localized primarily in the left ventrolateral prefrontal cortex (VLPFC), corresponding to Channels 14–16, 29, and 30, where the HC group exhibited a reduction in HbO from Session 1 to 2 (*p* < 0.05) and higher HbO levels than the MCI group in Session 1.

In the right DLPFC, a significant main effect of group was observed (*F* = 4.761, *p* = 0.037), suggesting differences in neural activation between the groups. However, post hoc comparisons did not yield statistically significant pairwise differences, indicating that while the overall trend differed between groups, the differences within specific sessions were not sufficient to reach significance. In contrast, a significant interaction effect between the group and session was found in the left VLPFC (*F* = 4.067, *p* = 0.023), as shown in Figures 6 and 7. Follow‐up analyses revealed that HC participants exhibited a significant reduction in HbO concentrations from Sessions 1 to 2 (*p* < 0.05), which may indicate improved neural efficiency through task repetition. Additionally, in Session 1, HC participants demonstrated significantly higher HbO levels than MCI participants (*p* < 0.05), suggesting a greater initial engagement of inhibitory control regions. Interestingly, within the MCI group, a significant decrease in the HbO concentration was observed from Sessions 2 to 3 (*p* < 0.05), although this did not follow the pattern of consistent cognitive adaptation typically observed in healthy aging. In the right VLPFC, a significant effect of time was observed (*F* = 5.072, *p* = 0.009). Post hoc analyses demonstrated that only the HC group exhibited significant decreases in HbO from Sessions 1 to 2 and 3 (*p* < 0.05), whereas the MCI group demonstrated no significant changes across sessions in this region. This suggests that the HC group experienced increased processing efficiency with repeated task exposure, whereas the MCI group did not demonstrate similar neurofunctional adaptations. No statistically significant main or interaction effects were found in the left DLPFC or in either the FPC or OFC. These results suggest that these regions were not differentially engaged across repeated sessions or between groups in the Stroop task. Taken together, these findings indicate that cognitively healthy older adults exhibit task‐related neural adaptation, particularly in the ventrolateral prefrontal regions, likely reflecting the improved efficiency of executive control mechanisms. In contrast, individuals with MCI demonstrated limited or inconsistent patterns of prefrontal modulation, suggesting reduced neural plasticity in response to repeated cognitive demands.

## 4 Discussion

This study aimed to compare behavioral performance and prefrontal cortical activation between cognitively healthy older adults and those with MCI during repeated execution of the Stroop task. By focusing on group differences in neural and behavioral adaptations, we sought to better understand selective inhibition and executive modulation during aging and cognitive decline.

The results revealed that with task repetition, healthy older adults exhibited significant reductions in reaction time across sessions, accompanied by a consistent decrease in HbO concentration within the VLPFC.

These behavioral improvements were complemented by a nonsignificant trend toward increased Stroop accuracy in the HC group, with Friedman analysis showing a main effect of time but Bonferroni‐corrected post hoc comparisons indicating only a tendency for Session 3 accuracy to be higher than in Sessions 1 and 2. These findings are more appropriately understood as co‐occurring trends, highlighting concurrent shifts in behavioral performance and neural activation over the course of task repetition. These findings suggest an enhanced processing efficiency and adaptive modulation of selective inhibition with repetition. In contrast, the MCI group demonstrated no significant behavioral improvements across sessions in either reaction time or accuracy and only limited changes in VLPFC activation. Notably, in the left VLPFC, a significant reduction in activation was observed only in the third session, whereas the right VLPFC did not demonstrate a consistent pattern of change. This convergence of neural and behavioral evidence suggests that the MCI group exhibits delayed or limited adaptation to repeated cognitive challenges, with deficits spanning both speed and accuracy domains. However, the current study design does not allow us to determine with certainty that such limitations are attributable to a specific neural mechanism.

The group‐by‐time interaction patterns highlight that, while healthy individuals may benefit from task repetition through cognitive adaptation and neurofunctional refinement, older adults with MCI demonstrate delayed or diminished neural plasticity. Specifically, while the healthy group demonstrated a decrease in neural activation as early as the second session, the MCI group exhibited delayed modulation, which became evident only between the second and third sessions. Accuracy improvements in the HC group followed a similar temporal pattern to these neural changes, supporting the interpretation that enhanced precision in performance is linked to more efficient neural resource allocation. This aligns with previous findings suggesting that executive dysfunction in MCI attenuates the emergence of practice effects (Davidson et al. 2003; Machulda et al. 2013; Traykov et al. 2007). The current results extend these observations by providing neurophysiological evidence that supports the notion of impaired adaptation in MCI. However, whether this delayed change is a unique feature of MCI or reflects the influence of other factors—such as age, sex, or differences in other cognitive domains—was not controlled for in the present study.

From a neuroimaging perspective, the observed reduction in VLPFC activation in the HC group was consistent with the results of fMRI studies in younger adults. For instance, Chen et al. (2013) reported decreased activation in the left DLPFC during repeated Stroop task performance, which was interpreted as reduced semantic conflict due to increased familiarity with the task. However, our findings diverged in that no significant DLPFC changes were observed in older adults, possibly reflecting age‐related decline in neural plasticity or cognitive flexibility, which may limit the expression of practice‐related efficiency gains in this region. Furthermore, while the results are reported at the level of Brodmann areas, it is important to acknowledge that the spatial resolution of fNIRS is inherently limited. Without MRI co‐registration, precise anatomical localization cannot be guaranteed. Therefore, these anatomical assignments should be regarded as approximations, and interpretations based on them should be made with appropriate caution.

Theoretically, these findings can be interpreted through the lens of an executive control framework. Aron (2011) distinguished between reactive and proactive control, emphasizing the critical role of the PFC, particularly the DLPFC. Braver (2012) further proposed that the DLPFC supports proactive control by maintaining goal‐relevant information in the working memory to modulate responses in advance. In contrast, the VLPFC has been linked more closely to selective inhibition, which is the process of suppressing task‐irrelevant information in real time. The observed reductions in VLPFC activation among healthy older adults suggest that improved task efficiency may result from reduced reliance on active inhibition mechanisms as the task becomes familiar, which is consistent with more effective selective inhibition. Importantly, the fact that accuracy improved alongside faster reaction times in the HC group indicates that this neural efficiency did not come at the expense of performance precision.

Despite the growing interest in neural markers of cognitive decline, current intervention studies on MCI frequently treat this population as a homogeneous group, with limited attention to functional subdomains. As Lim (2021) noted, neuroimaging‐based differentiation of specific cognitive processes in MCI remains underdeveloped. While the existing literature has demonstrated that physical exercise can enhance prefrontal activation and cognitive function in MCI (Hall et al. 2001; Li et al. 2011; Resnick and Spellbring 2000), its direct relationship with selective inhibition and VLPFC modulation remains unclear.

Furthermore, age and sex may interact to influence executive function. In particular, previous research has reported that women may be more susceptible to difficulties in emotion‐related executive processes (De Frias et al. 2006; Pua and Yu 2024). Such factors could offer a potential explanatory context for some of the variability observed in neural adaptation patterns within the MCI group. The current findings underscore the need for future research to delineate the subcomponents of executive function in MCI populations more precisely and employ neuroimaging tools for fine‐grained, function‐specific analysis.

### 4.1 Limitations

The present study has certain limitations. First, the sample size was relatively small, which may limit the generalizability of our findings. Future studies with larger and more diverse populations are required to validate and extend these results. Second, the gender distribution was markedly imbalanced between groups, with a substantially higher proportion of female participants in the MCI group. This imbalance may further limit the generalizability of the findings and could potentially obscure or confound gender‐related differences in neural and behavioral responses. Therefore, future research should aim to recruit more gender‐balanced samples to better account for potential sex‐related effects. Third, although participants were screened for general health and cognitive status, the present study did not statistically control for potential confounding variables such as age, years of education, depressive symptoms, or other cognitive domains (e.g., processing speed, language, and working memory) that are known to overlap with and influence Stroop performance. While the MoCA provides an index of global cognitive status, it does not offer domain‐specific assessments. The absence of such controls limits the ability to attribute the observed group differences specifically to inhibitory control, as disparities in these additional domains could have contributed to both behavioral and neural outcomes. Fourth, the fNIRS measurements were confined to the PFC, thereby excluding other brain regions that may play important roles in selective inhibition and cognitive control. Therefore, the observed neural dynamics may provide only a partial view of the broader neural network involved in task performance. Fifth, the use of a single cognitive task—the color–word Stroop task—to assess selective inhibition may not fully capture the complexity and variability of inhibitory control mechanisms. Employing a wider range of executive function tasks could allow cross‐validation of selective inhibition and better characterization of domain‐specific deficits. To build on the current findings, future research should incorporate multiple cognitive paradigms, extend neural recordings to additional cortical and subcortical regions, and consider multimodal imaging approaches. Furthermore, studies that include intervention strategies such as physical exercise or cognitive training, as well as longitudinal designs, may offer deeper insights into how repeated task engagement influences prefrontal adaptability and inhibitory control modulation in both healthy aging and cognitive decline.

The present study has certain limitations. First, the sample size was relatively small, which may limit the generalizability of our findings. Future studies with larger and more diverse populations are required to validate and extend these results. Second, the gender distribution between groups was notably imbalanced, with a substantially higher proportion of female participants in the MCI group. This imbalance may confound interpretations related to sex‐specific neural or behavioral differences and further restrict the generalizability of the outcomes. Future research should aim to recruit gender‐balanced samples to better account for potential sex‐related effects. Third, although participants were screened for general health and cognitive status, the study did not statistically control for potential confounding variables such as age, years of education, depressive symptoms, or performance in other cognitive domains (e.g., processing speed, language abilities, working memory), which are known to influence Stroop task performance. While the MoCA provides a useful index of global cognitive functioning, it does not offer domain‐specific insights. The lack of control over these variables limits the extent to which observed group differences can be attributed solely to inhibitory control, as overlapping deficits in other cognitive domains may have contributed to both behavioral and neural outcomes. Fourth, the fNIRS measurements were confined to the PFC, thereby excluding other brain regions that may play important roles in selective inhibition and cognitive control. Therefore, the observed neural dynamics may provide only a partial view of the broader neural network involved in task performance. Fifth, the use of a single cognitive task—the color–word Stroop task—to assess selective inhibition may not fully capture the complexity and variability of inhibitory control mechanisms. Employing a wider range of executive function tasks could allow cross‐validation of selective inhibition and better characterization of domain‐specific deficits. To build on the current findings, future research should incorporate multiple cognitive paradigms, extend neural recordings to additional cortical and subcortical regions, and consider multimodal imaging approaches. Furthermore, studies that include intervention strategies such as physical exercise or cognitive training, as well as longitudinal designs, may offer deeper insights into how repeated task engagement influences prefrontal adaptability and inhibitory control modulation in both healthy aging and cognitive decline.

### 4.2 Conclusions

This study investigated the neurobehavioral effects of repeated Stroop task performance in cognitively healthy older adults and individuals with MCI, with a particular focus on practice‐related changes in prefrontal cortical activation and the neurophysiological characteristics of selective inhibition. These findings demonstrate that healthy older adults exhibit both improved response times and a progressive decrease in activation of the left VLPFC across sessions, suggesting increased task efficiency and successful adaptation through repeated exposure. In contrast, participants with MCI demonstrated minimal changes in reaction time and only limited modulation of VLPFC activation, indicating a diminished practice effect and reduced capacity for dynamic inhibitory control. Notably, neither group exhibited significant changes in the left DLPFC, which may reflect an age‐related decline in neuroplasticity or cognitive flexibility, thereby limiting the potential for frontally mediated efficiency gain through repetition. These findings suggest that selective inhibition and the underlying prefrontal modulation differ substantially between cognitively healthy and MCI populations. Specifically, VLPFC adaptability may serve as a key neurophysiological marker for distinguishing between normal and impaired executive function in aging.

Overall, the results highlight the potential of combining repetition‐based cognitive paradigms such as the Stroop task with fNIRS to assess frontally mediated executive functions in aging populations. This methodological approach may contribute to the early detection of MCI and development of targeted intervention strategies. Future research should expand this work by incorporating diverse cognitive tasks and broader cortical regions, allowing for a more nuanced understanding of the subcomponents of executive dysfunction in MCI and their underlying neural mechanisms.

## Author Contributions

**Jae‐Hoon Lee**: conceptualization, methodology, validation, formal analysis, data curation, writing – original draft, visualization, funding acquisition, writing – review and editing. **Kyeongho Byun**: writing – original draft, writing – review and editing. **Minchul Lee**: data curation, writing – original draft, writing – review and editing. **Min‐Seong Ha**: conceptualization, validation, writing – original draft, project administration, supervision, writing – review and editing.

## Consent

Written informed consent was obtained from all study participants prior to enrollment. Additionally, written informed consent was obtained from all participants for the publication of this paper.

## Conflicts of Interest

The authors declare no conflicts of interest.

## Data Availability

Data supporting the findings of this study are available from the corresponding author upon request.
